# Muscle weakness post-COVID: a practical guide for primary care

**DOI:** 10.3399/bjgp24X740229

**Published:** 2024-11-29

**Authors:** Rebecca Payne, Tabitha Pring, Molly Hey, Gareth Payne, Trisha Greenhalgh

**Affiliations:** Clarendon-Reuben doctoral scholar, Nuffield Department of Primary Care Health Sciences, University of Oxford, Oxford; PhD student, Bangor University, Bangor.; Nuffield Department of Primary Health Care Sciences, University of Oxford, Oxford.; Nuffield Department of Primary Health Care Sciences, University of Oxford, Oxford.; Department of Clinical Neurophysiology, Betsi Cadwaldr University Health Board, Bangor, Gwynedd.; Nuffield Department of Primary Care Health Sciences, University of Oxford, Oxford.

## Introduction

Since the earliest stages of the pandemic, muscle weakness has been a key symptom described by patients post-COVID infection. Estimated to affect up to 60% of those with long COVID, it can have a profound effect on the ability to carry out activities of daily living.^1^ Many patients describe a fluctuating pattern to such symptoms, which can be triggered by exercise or fatigue.^2^ Most of these patients do not receive any muscle-specific investigations and many are told that they are experiencing the impacts of deconditioning or that they have a psychological or functional disorder. Such patients are often prescribed antidepressants or diverted down rehabilitation or psychological support pathways. Although some patients find this helpful, many patients experiencing muscle symptoms do not benefit from such approaches. Two case studies are given in Box 1.

**Box 1.** Case studies

Charlotte is a 45-year-old civil servant who caught COVID in summer 2023, approximately 18 months after her last COVID vaccination. During her initial illness she had fever, pronounced myalgia, fasciculations, and weakness, and struggled to lift her head off the pillow. As the acute illness resolved, she was left with ongoing muscle weakness, fasciculations, and brain fog. Her GP treated her with antidepressants, which were not effective, and referred her to a long COVID rehabilitation programme. She found advice about pacing and brain fog very helpful, but was unable to fully benefit from the programme because of the disparity in symptoms between herself and the other participants — while many others were focused on regaining their former fitness levels, she was struggling to sit up long enough to participate in the 2-hour sessions. A social encounter with the authors led to an EMG 6 months after the acute infection, which showed chronic neurogenic changes with marked polyphasia and fasciculations, suggesting damage to the motor neurones and some axon terminal resprouting in keeping with an attempt at reinnervation after the injury. There were no signs of acute denervation, such as fibrillations or positive sharp waves, implying there was no ongoing nerve damage occurring. The findings were consistent with a history of motor neuronopathy at the time of her COVID infection. The brain fog was most likely another manifestation of the same acute neurological injury. Liezel was a fit and healthy 43-year-old allied health professional when she contracted COVID in autumn 2020, prior to vaccination. She initially had symptoms of a viral URTI. During the following 2 months she noticed increased shortness of breath, tachycardia, and muscle weakness in her arms and legs, as well as severe brain fog and photophobia. She was unable to mobilise more than 2.5 m, or stand to carry out activities of daily living such as cooking. Because of a decreased ability to concentrate, she struggled with reading, socialising, and driving. During the following 4 months of graded exercises, she was able to walk twice daily a distance of 150 m at a time, but this left her with fluctuating fatigue levels. During the following 4 months her exercise tolerance continued to improve and she was able to mobilise 250 m consistently in one go, which she quantified as 40%. It was at this point that EMG was performed, 9 months post-onset, which showed normal nerve conduction study, no spontaneous activity at rest, chronic neurogenic changes on voluntary activity, and abnormal increased jitter. These findings are consistent with pathology in the nerve root or anterior cord. In light of the patient’s age, history of COVID infection, and symptoms, these pointed to myelopathy. (Although increased Jitter is classically associated with a neuromuscular junction disorder such as myasthenia gravis, it can be seen with other neuromuscular diseases too.) Shortly thereafter, she was diagnosed with thyroiditis and asthma, and with the treatment of levothyroxine and inhalers her functional level increased and plateaued at 60%. She returned to work, but struggled with muscle weakness and poor concentration. When she contracted COVID again, 18 months after the initial infection, it took 3 months for her function to stabilise. Shortly thereafter, HRT was prescribed and she rapidly progressed to 80%. She was then able to cope with employment demands, working 2 days a week. She used cycling, walking, and dancing to strengthen her leg muscles. Gradually she progressed to a 95% functional level at her 3-year recovery mark.

*EMG = electromyography. HRT = hormone replacement therapy. URTI = upper respiratory tract infection.*

As more is learned about the impact of COVID on the neuromuscular system, recent research has highlighted a variety of ways that COVID damages muscle and nerve cells,^3^ resulting in muscle weakness. This changing knowledge needs to lead to changing practice. It is therefore time to deploy Maya Angelou’s advice, *‘Do the best you can until you know better. Then when you know better, do better.’*

## What could be behind a patient’s muscle symptoms?

### Metabolic processes

COVID can damage the mitochondria and interfere with the electron transport chain supplying energy to muscles. This leads to fatigability, weakness, and reduced exercise tolerance,^4^ all of which can be exacerbated by poor sleep or nutrition, or intercurrent viral illnesses.^5^

### Motor nerve damage

During acute COVID infection, motor neurones and adjacent muscles can be directly damaged by the virus or by the immune response.^6^ Some patients will present with acute Guillain–Barré syndrome,^7^ whereas others experience prolonged muscle weakness. Regrowth of nerve fibres following such an event takes 2–3 years. These patients may be more likely to develop ‘brain fog’ symptoms as central nervous system tissues are also affected by COVID-related inflammation/immune responses.^8^

### Myelopathy

COVID can cause damage to the anterior spinal cord, resulting in myelopathy.^9^ This can present with a purely motor syndrome, with a mixture of upper and lower motor neurone features such as muscle wasting and brisk reflexes, with motor and sensory components, or with purely sensory symptoms (changes to the sensation of temperature and pain).

### Localised nerve damage

Specific plexopathies such as brachial neuritis and lumbosacral plexopathy have been reported following both COVID infection and vaccination.^10^^–^^12^ These disorders may be bilateral but are more commonly unilateral. Patients have discrete areas of motor and sensory axonal damage in an anatomically related pattern.

### COVID-induced myasthenia gravis

COVID is known to precipitate the onset of myasthenia gravis.^13^ Patients with this condition are likely to present with classic symptoms of ptosis and fatigability, with fluctuating muscle weakness.

### Non-COVID pathology

Other causes of muscle weakness are still common, including vitamin B and D deficiencies, and disorders such as polymyalgia rheumatica and polymyositis. Post-Intensive Care Unit patients are at high risk of neuropathies and myopathies.^14^

## What should the GP look for on clinical assessment? And will they find anything?

Taking a really careful history is key to differentiating ‘typical’ long COVID weakness from patients with other muscle pathologies triggered by a COVID-19 infection. A typical long COVID history involves an acute COVID-19 illness, followed by complete or partial recovery before the onset of symptoms such as ‘strange’, ‘energy-sapping’, and often fluctuating muscle fatigability and weakness.^15^

In the primary care setting, examination of patients with post-COVID muscle weakness should be targeted towards identifying myasthenic patients and assessing specific deficits such as brachial neuritis. The majority of other patients will have a normal examination, even if they have significant mitochondrial, muscle, or nerve damage.

The GP should check for myasthenia by looking for ptosis and undertaking an upward gaze test. If a patient develops ptosis or diplopia while sustaining an upward gaze (for 30–60 seconds), there is a significant chance of underlying myasthenia gravis.^16^

In patients with localised symptoms, the GP should perform a brief assessment of strength and sensation, concentrating on the affected areas. Although muscle wasting may coexist with weakness, it can be hard to identify.

Patients with a myelopathy may have muscle wasting, abnormal sensory findings, and brisk reflexes bilaterally, affecting either the lower limbs (bilaterally) or upper and lower limbs depending on the location of the affected area.

A normal physical examination does not exclude an organic cause for post-COVID muscle weakness.

## Who should the GP investigate?

Initial investigations should be targeted at identifying other causes that may be responsible for, or exacerbating, symptoms. These investigations are detailed in Box 2.

**Box 2.** Initial investigations for patients with muscle symptoms post-COVID

Creatinine kinase C-reactive protein Vitamin D B12 Folate Full blood count Thyroid function tests

Clinical judgement should guide further investigation and referral.^17^ Further investigations are often difficult to access in the UK setting unless a patient has evidence of a specific deficit such as in brachial neuritis or a well-recognised illness such as myasthenia gravis. Where available, electromyography (EMG), performed by Neurophysiology Departments, has a high diagnostic yield in these patients and often pinpoints the mechanism of muscle damage. However, it is not readily available across the UK. Patients may need to be referred to a neurologist in order to access EMG; a very clear history in the referral letter should be provided in order to avoid diversion to other services.

If it can be accessed, a validated questionnaire such as the C19-YRS (Covid-19 Yorkshire Rehabilitation Scale) or the (freely available) Bristol Activities of Daily Living Scale can yield useful information on the impact of symptoms on the patient’s quality of life.^18^ The time taken to complete these means it is likely to be impractical to complete within a GP appointment, but, where services have been commissioned locally, the patient can access via the ELAROS app and complete at home. Repeating 3-monthly is helpful for monitoring progress.

## How should the GP manage patients with significant muscle weakness post-COVID?

Establishing a therapeutic relationship is vital, particularly as many of these patients will have experienced minimising of their symptoms or inappropriate treatment pathways. Explaining the likely mechanisms of damage is highly valued by patients, as it validates their lived experience and gives them an explanation that they can share with others such as family members and employers. Explaining to patients that nerves will take 2–3 years to recover can be helpful in setting realistic expectations and encouraging hope.

Patients suspected of myasthenia gravis should be urgently referred using local pathways. Patients with a localised neuropathy should also be referred for further investigation. Where long COVID services exist, referral should be considered, but graded exercise programmes are not recommended.^19^^,^^20^ A detailed history should be taken and an examination performed in order to avoid inappropriately sending patients for rehabilitation who actually have an underlying neuromuscular disorder.

Modifiable causes of illness such as concomitant vitamin D deficiency should be identified and treated. B vitamins are essential for nerve regrowth, so, even in the absence of deficiency, supplementation should be considered.

Chronic disease of any type can lead to depression, and a sensitive conversation may be needed about mood, taking care that the patient does not end up feeling that their muscle symptoms are being dismissed, or misdiagnosed as depression or anxiety.

Patients should be supported to recognise and live within the limitations their muscle weakness places upon them — maintaining gentle levels of activity where possible, but avoiding ‘boom–bust’ cycles. As with any long-term condition, GP support for the patient needs to be holistic, going beyond the mere provision of information and encompassing elements such as signposting to sources of peer support, such as local long COVID groups, supporting the patient to manage their symptoms,^21^ and coordinating care.^22^ Patients may need to be signed off work for a period of time. Where additional support is needed for work, patients should be referred to occupational health services, or advised to seek support from services such as Citizens Advice. The group ‘Long Covid Kids’ has useful resources for young people and their families (https://www.longcovidkids.org/).

## Conclusion

COVID is known to cause muscle, nerve, and metabolic damage. Many patients presenting with post-COVID weakness, exertional symptoms, or fatigue will have identifiable defects on neurophysiological examination. When managing these patients, excluding treatable conditions such as myasthenia gravis, managing concomitant problems such as vitamin D deficiency, and explaining that improvement is likely over a period of 2–3 years can support the patient while nature heals what medicine can not.
